# Synthesis and structure of a square-planar copper–norflaxacin coordination complex

**DOI:** 10.1107/S205698902500787X

**Published:** 2025-09-09

**Authors:** Abdusamat Rasulov, Batirbay Torambetov, Jabbor Suyunov, Sadridin Eshkaraev, Laziza Kholmurodova, Bekzod Babamuratov, Jamshid Ashurov

**Affiliations:** a Termez University of Economics and Service, 41B Farovon St, Termiz, 190111, Uzbekistan; b National University of Uzbekistan named after Mirzo Ulugbek 4 University St Tashkent 100174 Uzbekistan; c Termez State University, Barkamol Avlod St 43, Termez, 190111, Uzbekistan; d Karshi State University, 17, Kuchabag street, Karshi City, 180119, Uzbekistan; e Institute of Bioorganic Chemistry, Academy of Sciences of Uzbekistan, M. Ulugbek St, 83, Tashkent, 100125, Uzbekistan; University of Aberdeen, United Kingdom

**Keywords:** crystal structure, norfloxacin, copper, zwitterion, Hirshfeld surface analysis

## Abstract

The title coordination complex, bis­[4-(3-carboxyl­ato-1-ethyl-6-fluoro-4-oxo-1,4-di­hydro­quinolin-7-yl)piperazin-1-ium-κ^2^*O*^3^,*O*^4^]copper(II) dinitrate, [Cu(C_16_H_18_FN_3_O_3_)_2_](NO_3_)_2_, was synthesized from norfloxacin (NF) and Cu(NO_3_)_2_·3H_2_O. The asymmetric unit contains half a mol­ecule of [Cu(NF)_2_] and one nitrate anion, with the copper atom located at a special crystallographic position with 1 site symmetry. The NF ligands are zwitterionic and coordinate to the metal atom *via* two oxygen atoms and the nitrate ions remain uncoordinated as counter-ions. The supra­molecular features include π–π stacking inter­actions as well as N—H⋯O and C—H⋯F hydrogen bonds and short [2.718 (3) Å] F⋯F inter­actions, which facilitate the formation of a columnar assembly parallel to the *b*-axis. Hirshfeld surface and two-dimensional fingerprint plot analysis were used to qu­antify the supra­molecular inter­actions of the compound.

## Abstract 2

The title complex adopts a square-planar geometry, involving the bidentate chelation of two norfloxacin mol­ecules with copper, while nitrate ions acts as counter-ions.

## 1. Chemical context

Norfloxacin (C_16_H_17_N_3_O_3_F; NF) is a synthetic fluoro­quinolone anti­biotic with broad-spectrum anti­bacterial activity, commonly used to treat various infectious diseases, particularly urinary tract and respiratory tract infections. It is well known for inhibiting bacterial DNA replication, making it effective against a wide range of pathogens (Holmes *et al.*, 1985 ▸; Goldstein *et al.*, 1987 ▸; Spencer *et al.*, 2023 ▸; Chongcharoen *et al.*, 2008 ▸; Mazuel, 1991 ▸; Marc *et al.*, 2019 ▸). NF predominantly exists in a zwitterionic form, in which the terminal nitro­gen atom of the piperazine ring is protonated by a hydrogen atom transferred from the carb­oxy­lic acid group (Rasulov *et al.*, 2024 ▸; Florence *et al.*, 2000 ▸; Barbas *et al.*, 2007 ▸). As a result of this, it can function as a bidentate ligand, capable of coordinating with metal ions through its carboxyl­ate (COO^−^) and keto (C=O) oxygen atoms (Tobón *et al.*, 2022 ▸). Metal ion coordination has been shown to enhance the activity of certain anti­biotics, and several studies have reported significant improvements in the pharmacological properties of fluoro­quinolones (Efthimiadou *et al.*, 2007 ▸; Turel, 2002 ▸). In this study, we report the synthesis, structure, Hirshfeld surface and fingerprint analysis of a coordination complex formed between norfloxacin and copper nitrate, [Cu(C_16_H_17_N_3_O_3_F)_2_](NO_3_)_2_ (**I**).
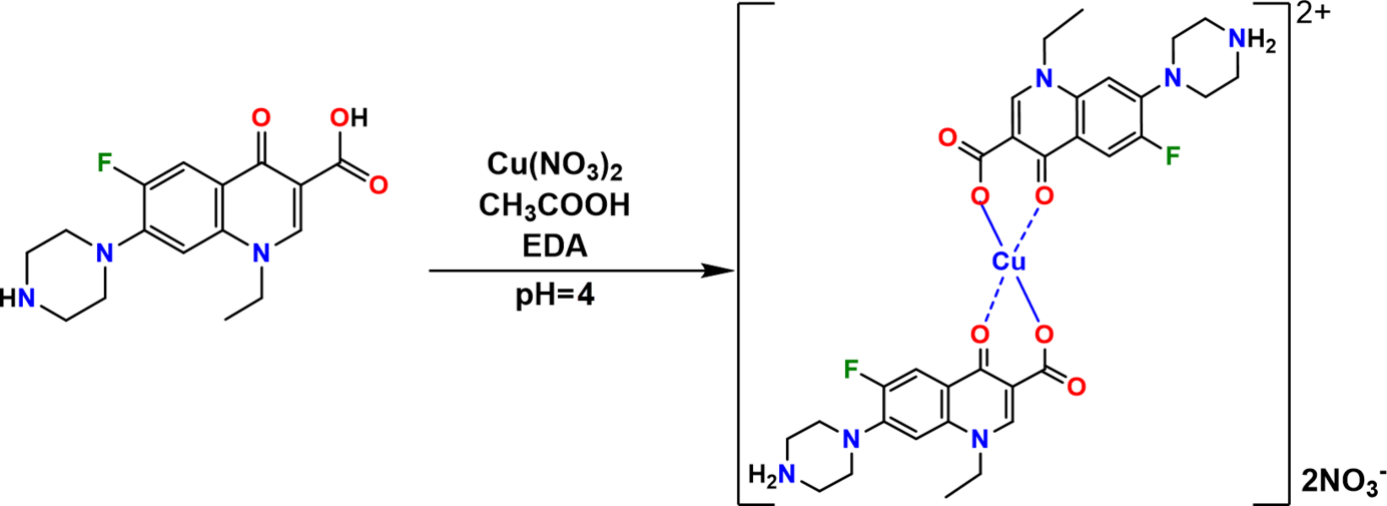


## 2. Structural commentary

**Figure 1:**
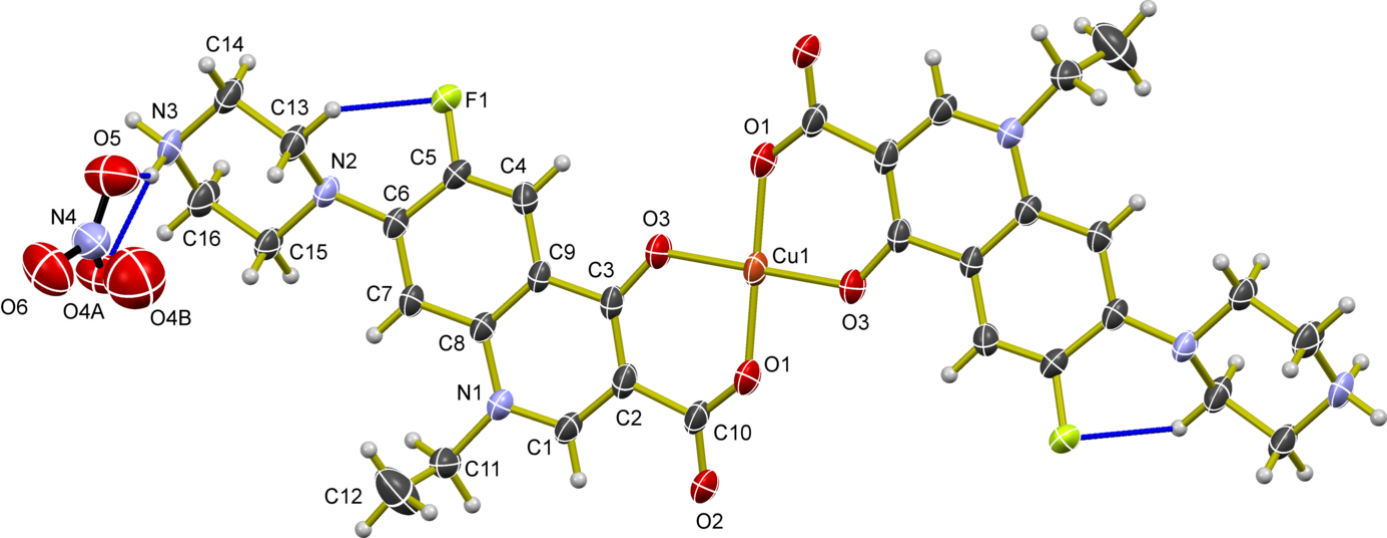
The mol­ecular structure of (**I**) drawn at the 50% ellipsoid probability level showing atom labeling (non-labelled atoms are generated by symmetry operation 2 − *x*, −*y*, −*z*). Hydrogen atoms are represented as small spheres with arbitrary radii and hydrogen bonds are indicated by dashed lines.

The asymmetric unit of (**I**) contains half of the mol­ecule of the [Cu(NF)_2_] complex along with one nitrate anion and the copper atom lies on a crystallographic inversion center. The terminal (secondary amine) nitro­gen atom (N3) within the piperazine ring is protonated by a proton formally transferred from the carb­oxy­lic acid group; hence, the NF ligand adopts a zwitterionic structure, featuring a negatively charged carboxyl­ate (–COO^−^) group at one end of the mol­ecule and a positively charged –NH_2_^+^ group on the terminal heterocyclic ring (Gunnam & Nangia, 2023 ▸). The copper (Cu^II^) atom is chelated by two norfloxacin ligands in a bidentate fashion through two oxygen atoms [carboxyl­ate oxygen atom O1 and the keto oxygen atom O3], resulting in a square planar complex with a coordination number of four. The Cu—O bond lengths range from 1.908 (2) to 1.928 (3) Å. The nitrate anion remains uncoordinated as a counter-ion (Fig. 1 ▸). An intra­molecular hydrogen bonding inter­action is observed between C13—H13*A* and F1 (Table 1 ▸), which generates an *S*(6) ring.

## 3. Supra­molecular features

**Figure 2:**
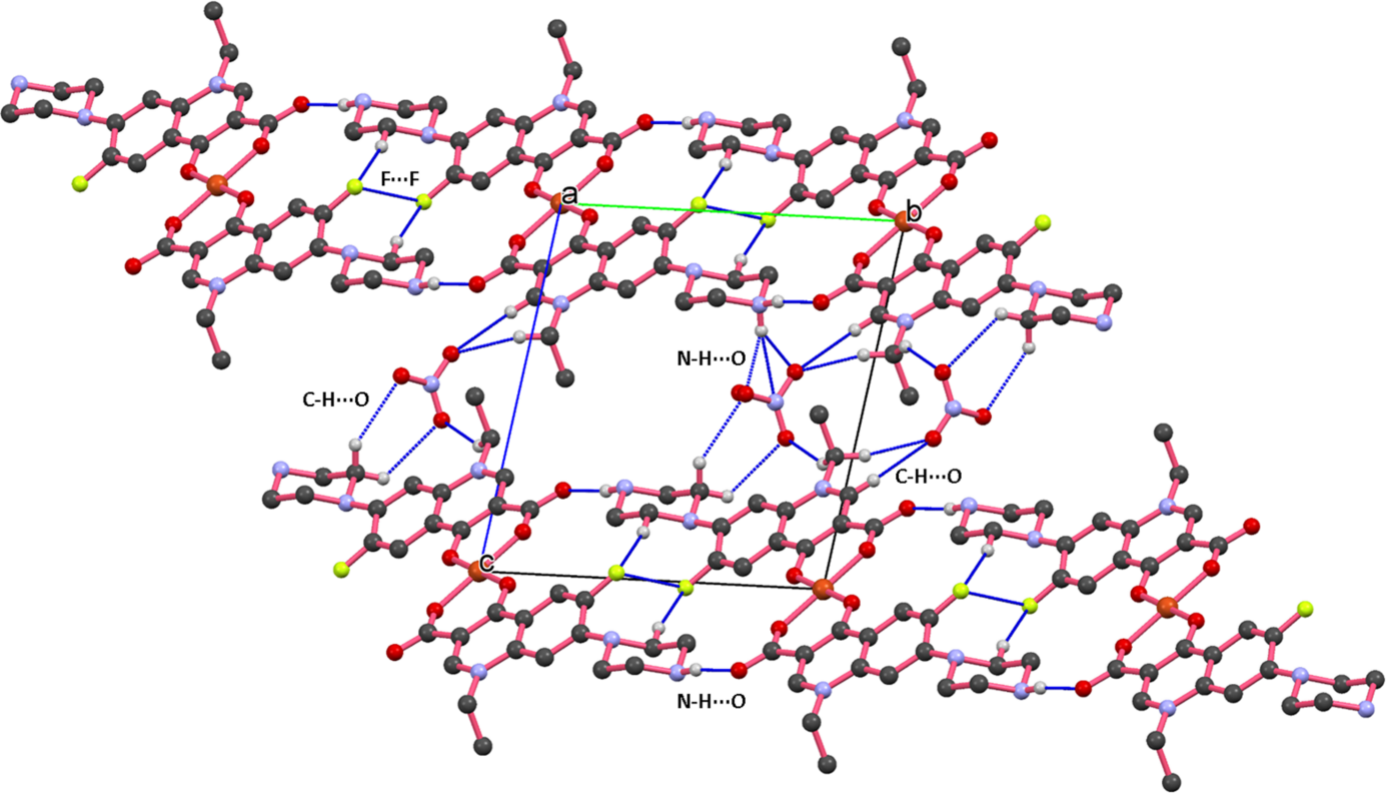
The packing of mol­ecules in (**I**) viewed along the *a-*axis direction, showing C—H⋯O, N—H⋯O and F⋯F inter­actions.

**Figure 3:**
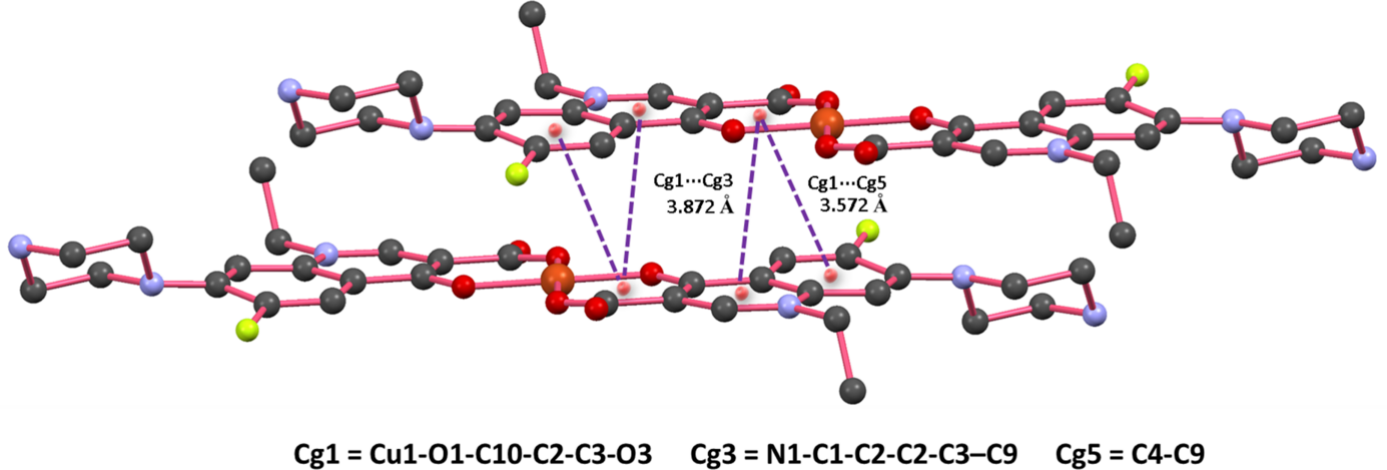
Visualization of the π–π stacking inter­actions between the mol­ecules of (**I**).

The extended structure of (**I**) features a number of inter­molecular inter­actions specifically, C—H⋯O, C—H⋯F, N—H⋯O, N—H⋯N and F⋯F (Table 1 ▸). The packing of mol­ecules when viewed down the *a* axis shows that the unit-translated mol­ecules are linked *via* N3—H3*A*⋯O2 (H⋯*A* = 1.82 Å), C13—H13*A*⋯F1 (2.42 Å) and short F⋯F [2.718 (3) Å] inter­actions, forming a columnar assembly propagating parallel to the to *b-*axis direction. The uncoordinated nitrate ion occupies the inter­stitial position between adjacent mol­ecular columns and participates in several hydrogen bonding inter­actions, including C—H⋯O, N—H⋯O and N—H⋯N contacts as illustrated in Fig. 2 ▸. Furthermore, strong π–π stacking inter­actions are observed between the quinoline ring system comprising the fused benzene ring (*Cg*5) and pyridine ring (*Cg*3) and a six-membered chelate ring (*Cg*1) formed by the coordination of norfloxacin’s oxygen atoms to the central copper atom. The centroid-to-centroid distances are measured to be 3.5724 (18) Å (*Cg*1⋯*Cg*5) and 3.8722 (18) Å (*Cg*1⋯*Cg*3), as illustrated in Fig. 3 ▸.

## 4. Hirshfeld surface analysis

**Figure 4:**
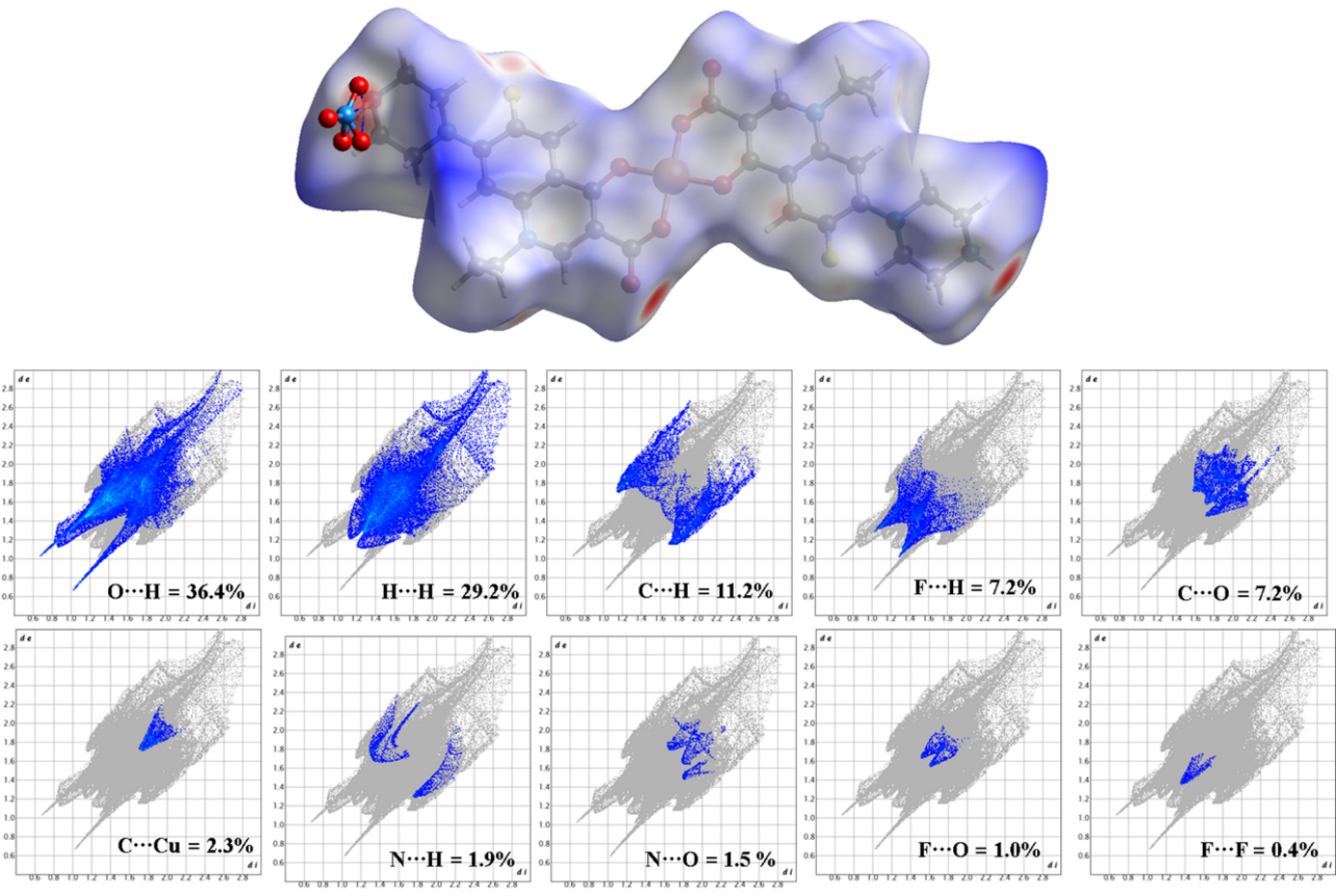
The Hirshfeld surface and fingerprint plots for (**I**).

To further qu­antify the inter­actions influencing the packing of (**I**), Hirshfeld surface analysis (Spackman & Jayatilaka, 2009 ▸) and two-dimensional fingerprint plot analysis (Spackman & McKinnon, 2002 ▸) were carried out using *CrystalExplorer21.5* (Spackman *et al.*, 2021 ▸). The two red spots on the Hishfeld surface area indicates close N—H⋯O contacts between adjacent mol­ecules, whereas other two lighter red spots represent C—H⋯F inter­actions. The fingerprint plots show the presence of O⋯H, H⋯H, C⋯H, F⋯H, C⋯O, C⋯Cu, N⋯H, N⋯O, F⋯O and F⋯F contributing 98.3% of the total inter­actions to the Hirshfeld surface area (Fig. 4 ▸).

## 5. Database survey

A survey conducted using the ConQuest software (CSD, Version 5.46, November 2024; Groom *et al.*, 2016 ▸) within the Cambridge Structural Database (CSD) identified over 26 crystal structures where NF acts as a bidentate ligand, coordinating through two oxygen atoms to metal centers such as Cr, Mn, Fe, Co, Ni, Cu, Zn, Mo, W, and Cd. These complexes predominantly adopt octa­hedral, square-pyramidal, and square-planar geometries. Among these, four copper complexes were found to exhibit a square-planar coordination, incorporating counter-ions including Cl^−^, ClO_4_^−^, SO_4_^2–^ and water mol­ecules of crystallization [CSD refcodes: XELNUZ (Živec *et al.*, 2012 ▸), WIFKEC (Ruíz *et al.*, 2007 ▸), SAFCUY (Xie *et al.*, 2004 ▸), and QECVAZ (Tobón Zapata *et al.*, 2022 ▸)]. Notably, no crystal structure was identified where a copper metal center forms a square-planar complex with a nitrate (NO_3_^−^) counter-ion.

## 6. Synthesis and crystallization

Because of the limited solubility of the ligand NF in water, the reaction was carried out in an acidic medium. An aqueous acidic solution with pH = 4 was prepared by mixing glacial acetic acid with 50 ml of water. A total of 0.200 mmol (0.0638 mg) of NF were dissolved in 5 ml of this solution to obtain a clear solution. Separately, 0.100 mmol (0.0242 mg) of Cu(NO_3_)_2_·3H_2_O were dissolved in water, yielding a clear blue solution. The two solutions were mixed in a 2:1 molar ratio and heated at 313 K with continuous stirring using a mechanical stirrer for 25–30 minutes. The resulting mixture remained turbid. To achieve a clear solution, ethyl­enedi­amine (EDA) was added dropwise with constant stirring until complete clarification was observed. The resulting clear solution was left to evaporate slowly at room temperature in a loosely covered vessel. After 10–12 days, blue block-shaped crystals of (**I**) suitable for X-ray diffraction analysis were obtained.

## 7. Refinement

Crystal data, data collection and structure refinement details are summarized in Table 2 ▸. Atom O4 of the nitrate anion was modelled as disordered over two adjacent sites of equal occupancy. H atoms were positioned geometrically (C—H = 0.93–0.97 Å) and refined as riding with *U*_iso_(H) = 1.2–1.5*U*_eq_(C).

## Supplementary Material

Crystal structure: contains datablock(s) I. DOI: 10.1107/S205698902500787X/hb8149sup1.cif

Structure factors: contains datablock(s) I. DOI: 10.1107/S205698902500787X/hb8149Isup2.hkl

CCDC reference: 2485024

Additional supporting information:  crystallographic information; 3D view; checkCIF report

## Figures and Tables

**Table 1:** Hydrogen-bond geometry (Å, °)

*D*—H⋯*A*	*D*—H	H⋯*A*	*D*⋯*A*	*D*—H⋯*A*
N3—H3*A*⋯O2^i^	0.89	1.82	2.698 (4)	170
N3—H3*B*⋯N4	0.89	2.52	3.406 (6)	173
N3—H3*B*⋯O5	0.89	2.11	2.887 (6)	145
N3—H3*B*⋯O4*A*	0.89	2.10	2.928 (9)	155
N3—H3*B*⋯O4*B*	0.89	2.27	3.115 (18)	158
C13—H13*A*⋯F1	0.97	2.27	2.888 (4)	121
C13—H13*A*⋯F1^ii^	0.97	2.42	3.379 (4)	168
C11—H11*A*⋯O5^iii^	0.97	2.40	3.295 (6)	153
C11—H11*B*⋯O6^iv^	0.97	2.57	3.420 (7)	146
C16—H16*A*⋯F1^v^	0.97	2.50	3.254 (5)	135

Symmetry codes: (i) 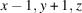; (ii) 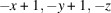; (iii) 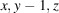; (iv) 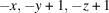; (v) 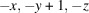.

**Table 2:** Experimental details

Crystal data	
Chemical formula	[Cu(C_16_H_17_FN_3_O_3_)_2_](NO_3_)_2_
*M*_r_	826.23
Crystal system, space group	Triclinic, *P* 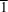
Temperature (K)	293
*a*, *b*, *c* (Å)	6.5956 (1), 11.2543 (2), 12.1236 (2)
α, β, γ (°)	99.303 (2), 90.783 (2), 100.118 (2)
*V* (Å^3^)	873.45 (3)
*Z*	1
Radiation type	Cu *K*α
μ (mm^−1^)	1.65
Crystal size (mm)	0.18 × 0.12 × 0.08

Data collection	
Diffractometer	XtaLAB Synergy, Single source at home/near, HyPix3000
Absorption correction	Multi-scan (*CrysAlis PRO*; Rigaku OD, 2020 ▸)
*T*_min_, *T*_max_	0.583, 1.000
No. of measured, independent and observed [*I* > 2σ(*I*)] reflections	7836, 3331, 2984
*R*_int_	0.028
(sin θ/λ)_max_ (Å^−1^)	0.614

Refinement	
*R*[*F*^2^ > 2σ(*F*^2^)], *wR*(*F*^2^), *S*	0.071, 0.232, 1.10
No. of reflections	3331
No. of parameters	260
No. of restraints	12
H-atom treatment	H-atom parameters constrained
Δρ_max_, Δρ_min_ (e Å^−3^)	1.95, −0.52

Computer programs: *CrysAlis PRO* (Rigaku OD, 2020 ▸), *SHELXT2014/5* (Sheldrick, 2015*a* ▸), *SHELXL2016/6* (Sheldrick, 2015*b* ▸) and *OLEX2* (Dolomanov *et al.*, 2009 ▸).

## supplementary crystallographic information

### Bis[4-(3-carboxylato-1-ethyl-6-fluoro-4-oxo-1,4-dihydroquinolin-7-yl)piperazin-1-ium-κ2O3,O4]copper(II) dinitrate Crystal data

**Table:**

[Cu(C_16_H_17_FN_3_O_3_)_2_](NO_3_)_2_	*Z* = 1
*M**_r_* = 826.23	*F*(000) = 427
Triclinic, *P*1	*D*_x_ = 1.571 Mg m^−^^3^
*a* = 6.5956 (1) Å	Cu *K*α radiation, λ = 1.54184 Å
*b* = 11.2543 (2) Å	Cell parameters from 4639 reflections
*c* = 12.1236 (2) Å	θ = 3.7–71.3°
α = 99.303 (2)°	µ = 1.65 mm^−^^1^
β = 90.783 (2)°	*T* = 293 K
γ = 100.118 (2)°	Needle, blue
*V* = 873.45 (3) Å^3^	0.18 × 0.12 × 0.08 mm

### Bis[4-(3-carboxylato-1-ethyl-6-fluoro-4-oxo-1,4-dihydroquinolin-7-yl)piperazin-1-ium-κ2O3,O4]copper(II) dinitrate Data collection

**Table:**

XtaLAB Synergy, Single source at home/near, HyPix3000 diffractometer	3331 independent reflections
Radiation source: micro-focus sealed X-ray tube, PhotonJet (Cu) X-ray Source	2984 reflections with *I* > 2σ(*I*)
Mirror monochromator	*R*_int_ = 0.028
Detector resolution: 10.0000 pixels mm^-1^	θ_max_ = 71.3°, θ_min_ = 7.3°
ω scans	*h* = −7→8
Absorption correction: multi-scan (CrysAlisPro; Rigaku OD, 2020)	*k* = −13→13
*T*_min_ = 0.583, *T*_max_ = 1.000	*l* = −12→14
7836 measured reflections	

### Bis[4-(3-carboxylato-1-ethyl-6-fluoro-4-oxo-1,4-dihydroquinolin-7-yl)piperazin-1-ium-κ2O3,O4]copper(II) dinitrate Refinement

**Table:**

Refinement on *F*^2^	Primary atom site location: dual
Least-squares matrix: full	Hydrogen site location: inferred from neighbouring sites
*R*[*F*^2^ > 2σ(*F*^2^)] = 0.071	H-atom parameters constrained
*wR*(*F*^2^) = 0.232	*w* = 1/[σ^2^(*F*_o_^2^) + (0.1596*P*)^2^ + 0.6898*P*] where *P* = (*F*_o_^2^ + 2*F*_c_^2^)/3
*S* = 1.10	(Δ/σ)_max_ < 0.001
3331 reflections	Δρ_max_ = 1.95 e Å^−^^3^
260 parameters	Δρ_min_ = −0.51 e Å^−^^3^
12 restraints	

### Bis[4-(3-carboxylato-1-ethyl-6-fluoro-4-oxo-1,4-dihydroquinolin-7-yl)piperazin-1-ium-κ2O3,O4]copper(II) dinitrate Special details

**Table:**

Geometry. All esds (except the esd in the dihedral angle between two l.s. planes) are estimated using the full covariance matrix. The cell esds are taken into account individually in the estimation of esds in distances, angles and torsion angles; correlations between esds in cell parameters are only used when they are defined by crystal symmetry. An approximate (isotropic) treatment of cell esds is used for estimating esds involving l.s. planes.

### Bis[4-(3-carboxylato-1-ethyl-6-fluoro-4-oxo-1,4-dihydroquinolin-7-yl)piperazin-1-ium-κ2O3,O4]copper(II) dinitrate Fractional atomic coordinates and isotropic or equivalent isotropic displacement parameters (Å^2^)

**Table:**

	*x*	*y*	*z*	*U*_iso_*/*U*_eq_	Occ. (<1)
Cu1	1.000000	0.000000	0.000000	0.0334 (3)	
F1	0.3549 (4)	0.3962 (2)	−0.01524 (18)	0.0431 (6)	
O1	0.9209 (4)	−0.1009 (2)	0.1121 (2)	0.0403 (6)	
O3	0.7835 (4)	0.0909 (2)	0.0324 (2)	0.0395 (6)	
O2	0.7146 (5)	−0.1896 (3)	0.2295 (2)	0.0502 (8)	
N1	0.3471 (5)	0.0646 (3)	0.2705 (2)	0.0337 (6)	
N2	0.1189 (5)	0.4249 (3)	0.1647 (2)	0.0332 (6)	
N3	−0.0609 (5)	0.6346 (3)	0.2447 (3)	0.0424 (8)	
H3A	−0.143875	0.686345	0.233616	0.051*	
H3B	−0.006627	0.656582	0.314222	0.051*	
C5	0.3682 (5)	0.3229 (3)	0.0627 (3)	0.0314 (7)	
C6	0.2366 (5)	0.3321 (3)	0.1528 (3)	0.0296 (7)	
C2	0.6306 (5)	−0.0116 (3)	0.1781 (3)	0.0331 (7)	
C3	0.6507 (5)	0.0771 (3)	0.1080 (3)	0.0298 (7)	
C7	0.2288 (5)	0.2438 (3)	0.2214 (3)	0.0320 (7)	
H7	0.136771	0.242568	0.278834	0.038*	
C9	0.5041 (5)	0.1603 (3)	0.1216 (3)	0.0284 (7)	
C8	0.3581 (5)	0.1568 (3)	0.2051 (3)	0.0293 (7)	
C4	0.5039 (5)	0.2450 (3)	0.0487 (3)	0.0325 (7)	
H4	0.596013	0.247626	−0.008592	0.039*	
N4	0.1658 (8)	0.7424 (4)	0.5042 (3)	0.0611 (11)	
O6	0.2118 (8)	0.7958 (4)	0.5992 (3)	0.0882 (13)	
C10	0.7633 (6)	−0.1071 (3)	0.1727 (3)	0.0351 (8)	
C13	0.2427 (6)	0.5494 (3)	0.1808 (3)	0.0389 (8)	
H13A	0.351210	0.552718	0.127553	0.047*	
H13B	0.306592	0.569652	0.255552	0.047*	
C15	−0.0439 (6)	0.4174 (3)	0.2464 (3)	0.0384 (8)	
H15A	0.017691	0.436025	0.321742	0.046*	
H15B	−0.123856	0.335089	0.235552	0.046*	
O5	0.2191 (8)	0.7803 (4)	0.4204 (3)	0.0846 (12)	
C1	0.4785 (6)	−0.0139 (3)	0.2550 (3)	0.0340 (7)	
H1	0.466345	−0.074724	0.299382	0.041*	
C11	0.1933 (6)	0.0519 (4)	0.3584 (3)	0.0431 (9)	
H11A	0.154466	−0.033762	0.365067	0.052*	
H11B	0.070471	0.080319	0.336570	0.052*	
C16	−0.1830 (6)	0.5077 (4)	0.2313 (4)	0.0465 (9)	
H16A	−0.250784	0.485830	0.157495	0.056*	
H16B	−0.288521	0.504248	0.286281	0.056*	
C14	0.1064 (7)	0.6421 (3)	0.1643 (4)	0.0433 (9)	
H14A	0.188015	0.723983	0.176487	0.052*	
H14B	0.047724	0.624597	0.088336	0.052*	
O4A	0.0084 (15)	0.6516 (8)	0.4863 (7)	0.069 (2)	0.5
C12	0.2773 (9)	0.1237 (7)	0.4689 (4)	0.0752 (16)	
H12A	0.309570	0.209013	0.463445	0.113*	
H12B	0.400008	0.096491	0.490200	0.113*	
H12C	0.176388	0.111645	0.524299	0.113*	
O4B	0.117 (3)	0.6338 (16)	0.4839 (14)	0.130 (5)	0.5

### Bis[4-(3-carboxylato-1-ethyl-6-fluoro-4-oxo-1,4-dihydroquinolin-7-yl)piperazin-1-ium-κ2O3,O4]copper(II) dinitrate Atomic displacement parameters (Å^2^)

**Table:**

	*U*^11^	*U*^22^	*U*^33^	*U*^12^	*U*^13^	*U*^23^
Cu1	0.0320 (4)	0.0337 (4)	0.0388 (5)	0.0191 (3)	0.0046 (3)	0.0038 (3)
F1	0.0552 (14)	0.0419 (11)	0.0431 (11)	0.0260 (10)	0.0126 (9)	0.0198 (9)
O1	0.0414 (14)	0.0406 (13)	0.0472 (14)	0.0270 (11)	0.0084 (11)	0.0101 (11)
O3	0.0381 (14)	0.0408 (14)	0.0474 (14)	0.0243 (11)	0.0132 (11)	0.0113 (11)
O2	0.0637 (19)	0.0473 (16)	0.0552 (16)	0.0397 (14)	0.0215 (14)	0.0213 (13)
N1	0.0366 (15)	0.0316 (14)	0.0379 (15)	0.0166 (12)	0.0061 (12)	0.0093 (11)
N2	0.0334 (15)	0.0279 (14)	0.0441 (16)	0.0167 (12)	0.0084 (12)	0.0101 (11)
N3	0.0478 (19)	0.0380 (16)	0.0501 (18)	0.0299 (14)	0.0080 (14)	0.0088 (14)
C5	0.0351 (17)	0.0262 (15)	0.0365 (16)	0.0128 (13)	0.0016 (13)	0.0082 (12)
C6	0.0278 (15)	0.0266 (15)	0.0371 (16)	0.0126 (12)	0.0011 (12)	0.0052 (12)
C2	0.0363 (18)	0.0299 (16)	0.0354 (16)	0.0175 (14)	−0.0014 (13)	−0.0005 (13)
C3	0.0288 (15)	0.0294 (16)	0.0328 (16)	0.0146 (13)	0.0011 (12)	−0.0004 (13)
C7	0.0322 (17)	0.0324 (16)	0.0358 (16)	0.0171 (13)	0.0051 (13)	0.0058 (13)
C9	0.0291 (16)	0.0249 (15)	0.0329 (16)	0.0127 (12)	0.0015 (12)	0.0012 (12)
C8	0.0297 (16)	0.0276 (15)	0.0338 (16)	0.0130 (12)	0.0016 (12)	0.0060 (12)
C4	0.0325 (17)	0.0312 (16)	0.0354 (16)	0.0117 (13)	0.0079 (13)	0.0036 (13)
N4	0.087 (3)	0.046 (2)	0.052 (2)	0.014 (2)	0.017 (2)	0.0098 (17)
O6	0.096 (3)	0.095 (3)	0.060 (2)	−0.002 (3)	0.008 (2)	−0.006 (2)
C10	0.0437 (19)	0.0309 (16)	0.0359 (17)	0.0223 (15)	0.0007 (14)	0.0039 (13)
C13	0.0357 (18)	0.0271 (16)	0.057 (2)	0.0142 (14)	0.0079 (15)	0.0076 (15)
C15	0.0341 (18)	0.0357 (17)	0.054 (2)	0.0209 (14)	0.0132 (15)	0.0171 (15)
O5	0.100 (3)	0.087 (3)	0.069 (2)	0.009 (2)	0.003 (2)	0.029 (2)
C1	0.0403 (19)	0.0294 (16)	0.0373 (17)	0.0181 (14)	0.0006 (14)	0.0073 (13)
C11	0.045 (2)	0.044 (2)	0.050 (2)	0.0212 (16)	0.0193 (16)	0.0207 (17)
C16	0.0354 (19)	0.044 (2)	0.070 (3)	0.0237 (17)	0.0134 (17)	0.0208 (19)
C14	0.048 (2)	0.0299 (17)	0.059 (2)	0.0208 (16)	0.0121 (17)	0.0124 (16)
O4A	0.075 (5)	0.068 (4)	0.057 (4)	−0.012 (4)	0.000 (3)	0.017 (3)
C12	0.065 (3)	0.118 (5)	0.048 (3)	0.027 (3)	0.019 (2)	0.014 (3)
O4B	0.134 (6)	0.126 (6)	0.127 (6)	0.020 (4)	0.009 (4)	0.020 (4)

### Bis[4-(3-carboxylato-1-ethyl-6-fluoro-4-oxo-1,4-dihydroquinolin-7-yl)piperazin-1-ium-κ2O3,O4]copper(II) dinitrate Geometric parameters (Å, º)

**Table:**

Cu1—O1^i^	1.928 (3)	C7—C8	1.400 (5)
Cu1—O1	1.928 (3)	C9—C8	1.407 (5)
Cu1—O3	1.908 (2)	C9—C4	1.400 (5)
Cu1—O3^i^	1.908 (2)	C4—H4	0.9300
F1—C5	1.362 (4)	N4—O6	1.218 (6)
O1—C10	1.279 (5)	N4—O5	1.197 (6)
O3—C3	1.285 (4)	N4—O4A	1.312 (9)
O2—C10	1.244 (5)	N4—O4B	1.192 (18)
N1—C8	1.398 (4)	C13—H13A	0.9700
N1—C1	1.337 (5)	C13—H13B	0.9700
N1—C11	1.488 (4)	C13—C14	1.526 (5)
N2—C6	1.398 (4)	C15—H15A	0.9700
N2—C13	1.474 (4)	C15—H15B	0.9700
N2—C15	1.472 (4)	C15—C16	1.513 (5)
N3—H3A	0.8900	C1—H1	0.9300
N3—H3B	0.8900	C11—H11A	0.9700
N3—C16	1.494 (5)	C11—H11B	0.9700
N3—C14	1.483 (5)	C11—C12	1.493 (7)
C5—C6	1.407 (5)	C16—H16A	0.9700
C5—C4	1.353 (5)	C16—H16B	0.9700
C6—C7	1.390 (5)	C14—H14A	0.9700
C2—C3	1.403 (5)	C14—H14B	0.9700
C2—C10	1.495 (4)	C12—H12A	0.9600
C2—C1	1.378 (5)	C12—H12B	0.9600
C3—C9	1.453 (4)	C12—H12C	0.9600
C7—H7	0.9300		
			
O1—Cu1—O1^i^	180.0	O5—N4—O4A	112.7 (5)
O3^i^—Cu1—O1	86.93 (11)	O4B—N4—O6	121.5 (9)
O3—Cu1—O1^i^	86.93 (11)	O4B—N4—O5	109.4 (9)
O3—Cu1—O1	93.07 (11)	O1—C10—C2	119.8 (3)
O3^i^—Cu1—O1^i^	93.07 (11)	O2—C10—O1	122.8 (3)
O3^i^—Cu1—O3	180.0	O2—C10—C2	117.3 (3)
C10—O1—Cu1	129.8 (2)	N2—C13—H13A	109.6
C3—O3—Cu1	126.3 (2)	N2—C13—H13B	109.6
C8—N1—C11	121.6 (3)	N2—C13—C14	110.2 (3)
C1—N1—C8	119.6 (3)	H13A—C13—H13B	108.1
C1—N1—C11	118.8 (3)	C14—C13—H13A	109.6
C6—N2—C13	113.9 (3)	C14—C13—H13B	109.6
C6—N2—C15	116.6 (3)	N2—C15—H15A	109.8
C15—N2—C13	110.4 (3)	N2—C15—H15B	109.8
H3A—N3—H3B	108.0	N2—C15—C16	109.4 (3)
C16—N3—H3A	109.4	H15A—C15—H15B	108.2
C16—N3—H3B	109.4	C16—C15—H15A	109.8
C14—N3—H3A	109.4	C16—C15—H15B	109.8
C14—N3—H3B	109.4	N1—C1—C2	124.9 (3)
C14—N3—C16	111.0 (3)	N1—C1—H1	117.5
F1—C5—C6	117.5 (3)	C2—C1—H1	117.5
C4—C5—F1	118.7 (3)	N1—C11—H11A	109.3
C4—C5—C6	123.8 (3)	N1—C11—H11B	109.3
N2—C6—C5	118.5 (3)	N1—C11—C12	111.4 (4)
C7—C6—N2	124.9 (3)	H11A—C11—H11B	108.0
C7—C6—C5	116.5 (3)	C12—C11—H11A	109.3
C3—C2—C10	123.5 (3)	C12—C11—H11B	109.3
C1—C2—C3	119.1 (3)	N3—C16—C15	110.4 (3)
C1—C2—C10	117.4 (3)	N3—C16—H16A	109.6
O3—C3—C2	126.5 (3)	N3—C16—H16B	109.6
O3—C3—C9	117.1 (3)	C15—C16—H16A	109.6
C2—C3—C9	116.4 (3)	C15—C16—H16B	109.6
C6—C7—H7	119.6	H16A—C16—H16B	108.1
C6—C7—C8	120.7 (3)	N3—C14—C13	109.1 (3)
C8—C7—H7	119.6	N3—C14—H14A	109.9
C8—C9—C3	121.7 (3)	N3—C14—H14B	109.9
C4—C9—C3	119.7 (3)	C13—C14—H14A	109.9
C4—C9—C8	118.6 (3)	C13—C14—H14B	109.9
N1—C8—C7	121.5 (3)	H14A—C14—H14B	108.3
N1—C8—C9	118.0 (3)	C11—C12—H12A	109.5
C7—C8—C9	120.5 (3)	C11—C12—H12B	109.5
C5—C4—C9	119.3 (3)	C11—C12—H12C	109.5
C5—C4—H4	120.4	H12A—C12—H12B	109.5
C9—C4—H4	120.4	H12A—C12—H12C	109.5
O6—N4—O4A	119.4 (5)	H12B—C12—H12C	109.5
O5—N4—O6	125.6 (5)		
			
Cu1—O1—C10—O2	168.6 (3)	C8—N1—C11—C12	−90.5 (5)
Cu1—O1—C10—C2	−12.3 (5)	C8—C9—C4—C5	1.9 (5)
Cu1—O3—C3—C2	−3.7 (5)	C4—C5—C6—N2	173.3 (3)
Cu1—O3—C3—C9	177.3 (2)	C4—C5—C6—C7	−9.1 (5)
F1—C5—C6—N2	−7.5 (5)	C4—C9—C8—N1	174.2 (3)
F1—C5—C6—C7	170.2 (3)	C4—C9—C8—C7	−6.0 (5)
F1—C5—C4—C9	−173.4 (3)	C10—C2—C3—O3	−1.2 (5)
O3—C3—C9—C8	−177.2 (3)	C10—C2—C3—C9	177.8 (3)
O3—C3—C9—C4	4.2 (5)	C10—C2—C1—N1	180.0 (3)
N2—C6—C7—C8	−177.9 (3)	C13—N2—C6—C5	−60.8 (4)
N2—C13—C14—N3	58.3 (4)	C13—N2—C6—C7	121.7 (4)
N2—C15—C16—N3	−57.9 (4)	C13—N2—C15—C16	59.8 (4)
C5—C6—C7—C8	4.6 (5)	C15—N2—C6—C5	168.8 (3)
C6—N2—C13—C14	166.1 (3)	C15—N2—C6—C7	−8.6 (5)
C6—N2—C15—C16	−168.2 (3)	C15—N2—C13—C14	−60.5 (4)
C6—C5—C4—C9	5.8 (5)	C1—N1—C8—C7	−177.5 (3)
C6—C7—C8—N1	−177.6 (3)	C1—N1—C8—C9	2.3 (5)
C6—C7—C8—C9	2.6 (5)	C1—N1—C11—C12	89.0 (5)
C2—C3—C9—C8	3.7 (5)	C1—C2—C3—O3	−179.8 (3)
C2—C3—C9—C4	−174.9 (3)	C1—C2—C3—C9	−0.8 (5)
C3—C2—C10—O1	9.3 (5)	C1—C2—C10—O1	−172.1 (3)
C3—C2—C10—O2	−171.5 (3)	C1—C2—C10—O2	7.1 (5)
C3—C2—C1—N1	−1.4 (5)	C11—N1—C8—C7	2.0 (5)
C3—C9—C8—N1	−4.5 (5)	C11—N1—C8—C9	−178.2 (3)
C3—C9—C8—C7	175.3 (3)	C11—N1—C1—C2	−178.9 (3)
C3—C9—C4—C5	−179.4 (3)	C16—N3—C14—C13	−56.8 (4)
C8—N1—C1—C2	0.6 (5)	C14—N3—C16—C15	57.4 (4)

Symmetry code: (i) −*x*+2, −*y*, −*z*.

### Bis[4-(3-carboxylato-1-ethyl-6-fluoro-4-oxo-1,4-dihydroquinolin-7-yl)piperazin-1-ium-κ2O3,O4]copper(II) dinitrate Hydrogen-bond geometry (Å, º)

**Table:**

*D*—H···*A*	*D*—H	H···*A*	*D*···*A*	*D*—H···*A*
N3—H3*A*···O2^ii^	0.89	1.82	2.698 (4)	170
N3—H3*B*···N4	0.89	2.52	3.406 (6)	173
N3—H3*B*···O5	0.89	2.11	2.887 (6)	145
N3—H3*B*···O4*A*	0.89	2.10	2.928 (9)	155
N3—H3*B*···O4*B*	0.89	2.27	3.115 (18)	158
C13—H13*A*···F1	0.97	2.27	2.888 (4)	121
C13—H13*A*···F1^iii^	0.97	2.42	3.379 (4)	168
C11—H11*A*···O5^iv^	0.97	2.40	3.295 (6)	153
C11—H11*B*···O6^v^	0.97	2.57	3.420 (7)	146
C16—H16*A*···F1^vi^	0.97	2.50	3.254 (5)	135

Symmetry codes: (ii) *x*−1, *y*+1, *z*; (iii) −*x*+1, −*y*+1, −*z*; (iv) *x*, *y*−1, *z*; (v) −*x*, −*y*+1, −*z*+1; (vi) −*x*, −*y*+1, −*z*.
